# Beyond White-Light Colonoscopy: Water Exchange, Image-Enhanced Endoscopy, and Artificial Intelligence for Colorectal Neoplasia Detection—From Single Tools to Combined Strategies: A Scoping Review

**DOI:** 10.3390/cancers18213553

**Published:** 2026-11-05

**Authors:** Teodora Spataru, Remus Popescu, Alina Pistritu, Marina Cozma, Lucian Negreanu

**Affiliations:** 1 Internal Medicine Department, Carol Davila University of Medicine and Pharmacy, 020021 Bucharest, Romania; spataru.teodora93@gmail.com (T.S.); alina.pistritu93@gmail.com (A.P.); cozmamarina97@gmail.com (M.C.); negreanu_99@yahoo.com (L.N.); 2 Gastroenterology Department, University Emergency Hospital of Bucharest, 020021 Bucharest, Romania

**Keywords:** colonoscopy, adenoma detection rate, water exchange, image-enhanced endoscopy, linked color imaging, computer-aided detection, artificial intelligence, scoping review

## Abstract

**Background:** Water exchange, image-enhanced endoscopy (IEE), and computer-aided detection (CADe) address different reasons why colorectal lesions may be missed. Whether their benefits add up when the techniques are combined remains unclear. **Objective:** We mapped clinical and video-based evidence on combinations of these techniques for colorectal neoplasia detection. **Methods:** We conducted a scoping review following JBI methodology and PRISMA-ScR, with a protocol registered on the Open Science Framework. We searched PubMed, Web of Science, Scopus, ClinicalTrials.gov, and reference lists through September 2026. Eligible studies evaluated at least two techniques together in adults and reported a detection outcome. Large language model tools assisted screening, with reviewer verification. We summarized findings descriptively and added a structured, domain-based critical appraisal during revision. **Results:** From 644 database records, we included 18 studies: 13 patient-level clinical studies, nine randomized, and five lesion- or video-level studies. Most examined IEE with CADe. One trial directly added CADe to water exchange; one tested all three technique groups. No included clinical study used water immersion. Randomized incremental comparisons showed inconsistent adenoma detection rate (ADR) gains, while multicomponent trials could not isolate component effects. Historical controls, discretionary technique selection, selected video material, and incomplete reporting constrained other comparisons. Evidence on advanced and serrated lesions, procedural burden, and patient-important outcomes was limited. **Conclusions:** Combined strategies improved detection in some settings, but comparative effectiveness and net clinical benefit remain uncertain. Trials should isolate component effects and measure clinically relevant detection alongside procedural burden.

## Simple Summary

Colonoscopy can prevent colorectal cancer by finding and removing growths before they become cancerous, but some lesions are missed. Several techniques aim to improve detection: water can clean the colon during the examination, enhanced imaging can make lesions easier to see, and artificial intelligence can highlight suspicious areas. We reviewed studies that used at least two of these approaches together to see which combinations have been tested and what benefits or drawbacks were reported. We found 18 studies. Some combinations improved detection, but the results varied, and few studies showed which technique was responsible for the improvement. Evidence about more serious lesions, extra time and unnecessary removals was limited. Our review identifies what future studies need to measure before combined approaches can be recommended more widely.

## 1. Introduction

Colonoscopy reduces the incidence and mortality from colorectal cancer (CRC) by detecting and removing precancerous lesions. However, its protective effect depends on the quality of the examination. The adenoma detection rate (ADR), defined as the proportion of colonoscopies in which at least one adenoma is identified, is the primary indicator of quality. Each 1% increase in the ADR has been associated with a 3% reduction in the risk of interval CRC and a 5% reduction in the risk of death from CRC [1]. However, the ADR varies considerably among endoscopists, ranging from 7.4% to 52.5% even within the same healthcare system [1].

A significant proportion of lesions remain undetected during the initial examination. Studies using tandem colonoscopies have estimated miss rates of approximately 26% for adenomas, 9% for advanced adenomas, and 27% for serrated polyps [2]. Missed lesions are often small, flat, located in the proximal colon, or serrated, and suboptimal bowel preparation further reduces the likelihood of their detection [2].

Several techniques aim to address these limitations at different stages of colonoscopy. Water exchange (WE) removes residual fecal matter during insertion and reduces colonic distension, so that the mucosa is cleaner for inspection during withdrawal [3]. Image-enhanced endoscopy (IEE), which includes chromoendoscopy with dyes and electronic modes such as narrow-band imaging (NBI), linked color imaging (LCI), blue light imaging (BLI), texture and color enhancement imaging (TXI), and i-scan, increases the contrast between lesions and the surrounding mucosa [4]. Computer-aided detection (CADe), based on artificial intelligence, alerts the endoscopist in real time to suspicious lesions in the field of view.

Several of these techniques have improved ADR in randomized trials, although the magnitude of the benefit varies depending on the technique and the outcome assessed. A network meta-analysis of 17 studies, involving 10,350 patients, showed that WE increased ADR compared with air insufflation [5]. In a larger network meta-analysis comprising 124 studies, WE, i-scan, LCI, and CADe improved the ADR compared with standard colonoscopy. None of the interventions evaluated showed a statistically significant improvement in advanced adenoma detection, while only NBI improved the detection of serrated lesions [6]. Regarding CADe, the American Gastroenterological Association acknowledged the increase in ADR but was unable to make a recommendation for or against routine use, given the very low certainty of the evidence regarding outcomes important to patients, including the incidence of CRC and the burden of additional surveillance [7].

Because these techniques are performed at different stages of the examination—cleaning during insertion, visualization during withdrawal, and lesion detection within the field of view—their effects may be complementary [3,8,9]. WE could reduce CADe alerts triggered by fecal debris and air bubbles, while IEE could make subtle lesions more evident to both the endoscopist and the algorithm. However, these potential advantages require direct testing. The performance of a CADe system may depend on the imaging modality used, and the benefit of adding a technique may vary depending on the endoscopist’s baseline detection performance. Additional tools may also increase withdrawal time, the number of false-positive alerts, or the resection of non-neoplastic lesions. Network meta-analyses compare individual interventions, often indirectly [6,10,11], but cannot determine the incremental benefit of using them together.

Studies that directly evaluate combined strategies have emerged recently. Some analyze the addition of one technique to another, while others compare a bundle of techniques with standard colonoscopy. The evidence also includes observational studies and analyses of recorded procedures, with significant differences among comparators and the outcomes assessed. Previous reviews on the combination of WE with artificial intelligence were published before randomized trials on this combination became available [3,8]. We did not identify any review that maps the clinical and video-based evidence for all three groups of techniques while also distinguishing between incremental comparisons and multi-component strategies. Therefore, a scoping review is appropriate to describe the available evidence and identify those comparisons that have not yet been tested.

The purpose of this scoping review is to map the evidence regarding the combined use of water-aided colonoscopy, IEE, and CADe for the detection of colorectal neoplasia in adults undergoing colonoscopy for screening, following a positive fecal test, for surveillance after polypectomy, or for routine diagnostic indications. We considered the concurrent use of at least two of these techniques, both in clinical studies and in analyses of recorded procedures. The review questions were:

1. Which combinations of water-aided colonoscopy, IEE, and CADe have been evaluated, and in what types of studies?
2. Which studies evaluate the incremental effect of adding one technique to another, and which compare a multi-component strategy with standard colonoscopy?
3. What detection outcomes were reported beyond ADR, including the number of adenomas per colonoscopy, miss rates, serrated and advanced lesions, and lesions in the proximal colon?
4. What aspects related to procedural burden have been reported, including withdrawal time, false-positive alerts, and resection of non-neoplastic lesions?
5. Is there evidence that the observed benefit varies depending on baseline detection performance or the endoscopist’s experience?
6. Which combinations have not yet been tested or are being evaluated in registered studies?

## 2. Materials and Methods

### 2.1. Protocol and Registration

We conducted this scoping review according to the JBI methodology for this type of synthesis [12] and report it in accordance with the PRISMA extension for scoping reviews (PRISMA-ScR) [13]. We registered the protocol on the Open Science Framework on 26 September 2026 (https://osf.io/upqjm; https://doi.org/10.17605/OSF.IO/UPQJM). The MEDLINE search and the assessment of the records identified through it had been completed prior to registration, as specified in the protocol. We describe deviations from the protocol in Section 2.7.

### 2.2. Eligibility Criteria

We defined eligibility using the population, concept, and context framework [12].

Population: We considered studies that included adults undergoing colonoscopy for screening, following a positive fecal immunochemical test or fecal occult blood test, for surveillance, or for routine diagnostic indications. We excluded studies limited to the surveillance of inflammatory bowel disease or hereditary colorectal cancer syndromes.

Concept: Studies had to use at least two of the following techniques during the same colonoscopy: water-aided colonoscopy (water exchange or water immersion); image-enhanced endoscopy (IEE), including dye-based chromoendoscopy, narrow-band imaging (NBI), linked color imaging (LCI), blue light imaging (BLI), texture and color enhancement imaging (TXI), i-scan, or an equivalent modality; and computer-aided detection (CADe). Studies were also required to report a detection outcome, such as the adenoma detection rate, the number of adenomas per colonoscopy, the lesion miss rate, or the recognition of individual lesions. We excluded studies of a single technique, direct comparisons between two techniques where they were not used together, and studies in which artificial intelligence was used exclusively for lesion characterization or histologic prediction.

Water-aided colonoscopy is used here as an umbrella term. In WE, infused water and residual contents are removed predominantly during insertion; in water immersion, most infused water is removed during withdrawal [3,8]. These methods are recorded separately and are not treated as interchangeable.

Context: We considered studies from any clinical setting and any country. Eligible sources included randomized and non-randomized clinical studies, analyses of colonoscopy video recordings, and abstracts presented at conferences, provided they offered data that could be extracted. We identified studies that were registered but had no available results and reported them separately. We excluded studies on the development of algorithms based on static images, reviews, editorials, letters, and guidelines. To be eligible for evaluation, a publication had to have the full text or abstract available in English.

### 2.3. Information Sources and Search Strategy

We conducted a search in MEDLINE via PubMed on 25 September 2026, followed by two additional searches on 26 September 2026. We searched the Web of Science Core Collection (advanced search, “topic” field) and Scopus (“title,” “abstract,” and “keywords” fields) on 26 September 2026. On the same day, we also searched ClinicalTrials.gov. We also checked the reference lists of the included articles that were available in full text.

The search strategy combined three groups of terms—water-aided colonoscopy, IEE, and CADe—and included terms related to colonoscopy and colorectal neoplasia. We did not apply any date or language restrictions during the search phase. Supplementary Table S1 presents the available search strings, search dates, and the number of records retrieved from each source.

### 2.4. Selection of Evidence Sources

We removed duplicate records based on digital object identifiers (DOIs) and normalized titles. For the main PubMed search, Claude Opus 5.5 (Anthropic, San Francisco, CA, USA) and ChatGPT (OpenAI) independently assessed the titles and abstracts against the eligibility criteria. Two evaluators (T.S. and R.P.) then independently reviewed each record retained or classified as uncertain by either of the two tools, including all cases where their classifications differed. The two evaluators also reviewed the titles of all records excluded by the tools; they did not identify any additional eligible studies in this process.

For Web of Science, Scopus, and supplementary searches, the initial screening was performed using Claude. Both reviewers independently reviewed the records proposed for inclusion or classified as uncertain; records excluded by the tool were re-evaluated by both reviewers. The exports from Web of Science and Scopus did not contain abstracts, so we searched for them in PubMed or on the publisher’s website when the title was insufficient to determine eligibility.

**Figure 1:**
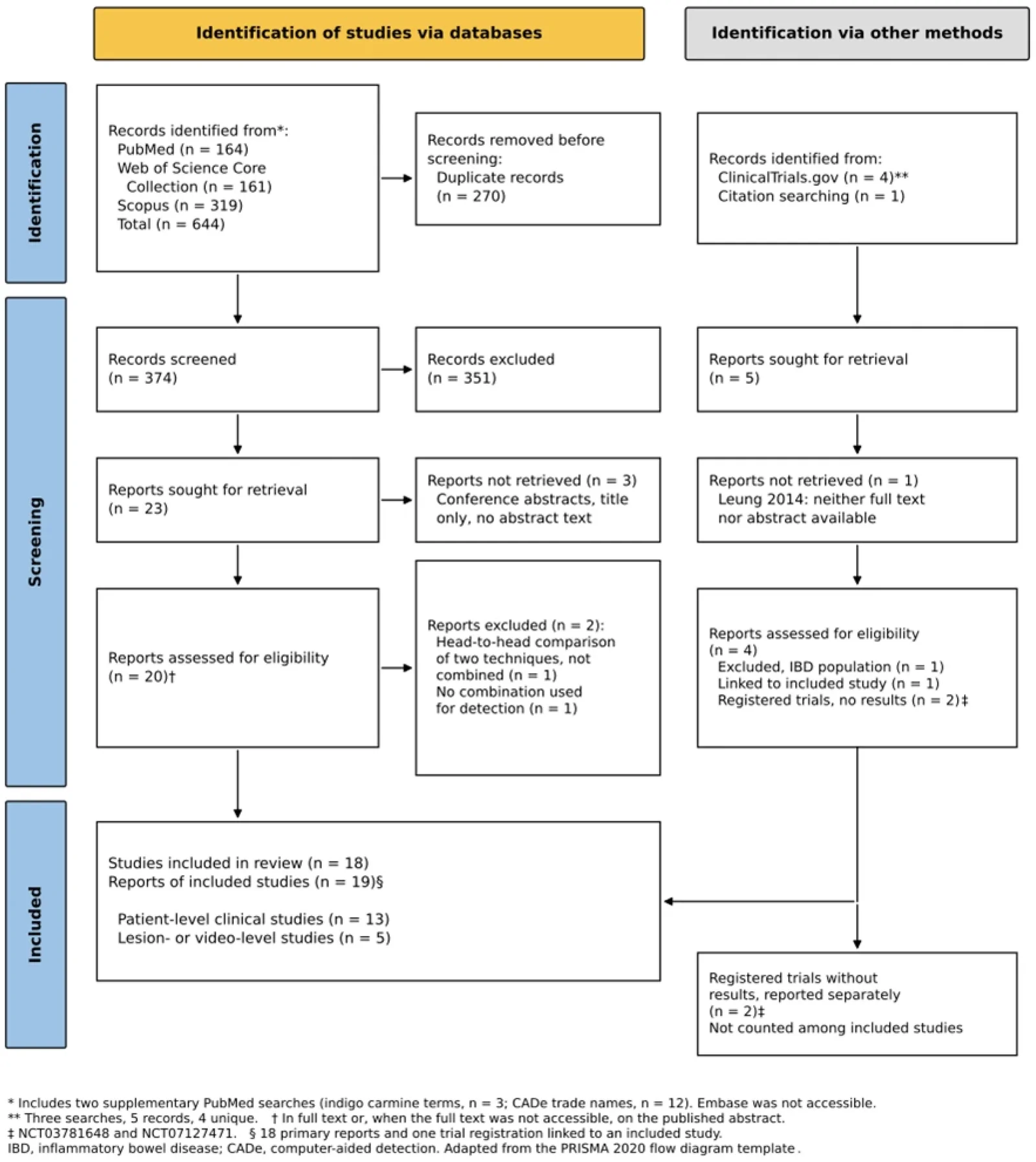
PRISMA flow diagram of study selection [15].

We assessed the full text of potentially eligible publications when available. Four studies were initially assessed from their published abstracts. During revision, we obtained the full text of Leung 2011 [14] and checked its design and results against the report, leaving three included studies with abstract-only data. We resolved disagreements through discussion and recorded decisions and their reasons in a decision log. Figure 1 illustrates the selection process in a flowchart adapted from the PRISMA 2020 template [15].

### 2.5. Data Extraction and Organization

We used the data collection form defined in the protocol. Data were extracted from full texts or, for the three studies whose full texts remained inaccessible, from published abstracts. The full text of Leung 2011 [14], obtained during revision, replaced the abstract as the charting source. We linked reports of the same study, such as a trial registration and its publication, and treated them as a single study. A large language model tool assisted data extraction; both reviewers independently verified the originally charted information against the available source reports.

For each study, we recorded the design, country, number of centers, and study registration; the study population and endoscopists; the combined techniques, imaging modality, and CADe system; as well as the level at which the outcomes were measured: patient, lesion, or video recording. We also recorded the type of comparison, the definitions and results of detection metrics, withdrawal time, false-positive alerts, resection of non-neoplastic lesions, and the limitations mentioned by the study authors.

### 2.6. Critical Appraisal and Synthesis of Results

During revision, we added a structured critical appraisal to help distinguish findings supported by randomized comparisons from those affected by confounding, selection, or limited reporting. We used a review-specific domain framework covering selection and allocation; comparability, confounding, and co-interventions; outcome assessment; and completeness and reporting. For lesion- and video-level studies, we also considered selection of known lesions, training and testing conditions, and whether the outcome represented live clinical detection. We recorded supporting methods, concerns, and information that could not be assessed in Supplementary Table S2, with source locators. This was a domain-based critical appraisal, not an application of a validated risk-of-bias instrument; we assigned no numerical score or overall low/high risk category. We considered applicability separately from internal validity, so single-centre or single-operator design did not by itself determine bias.

We summarized findings descriptively and kept patient-level clinical outcomes separate from lesion- and video-level performance. Within the clinical studies, we distinguished randomized incremental comparisons, randomized multicomponent strategies, non-randomized comparisons, and observational analyses of discretionary technique use. We interpreted findings in light of the methodological concerns in Supplementary Table S2 rather than treating these designs as equivalent. We did not rank combinations or pool results statistically because the interventions, comparators, populations, and outcomes differed substantially.

**Figure 2:**
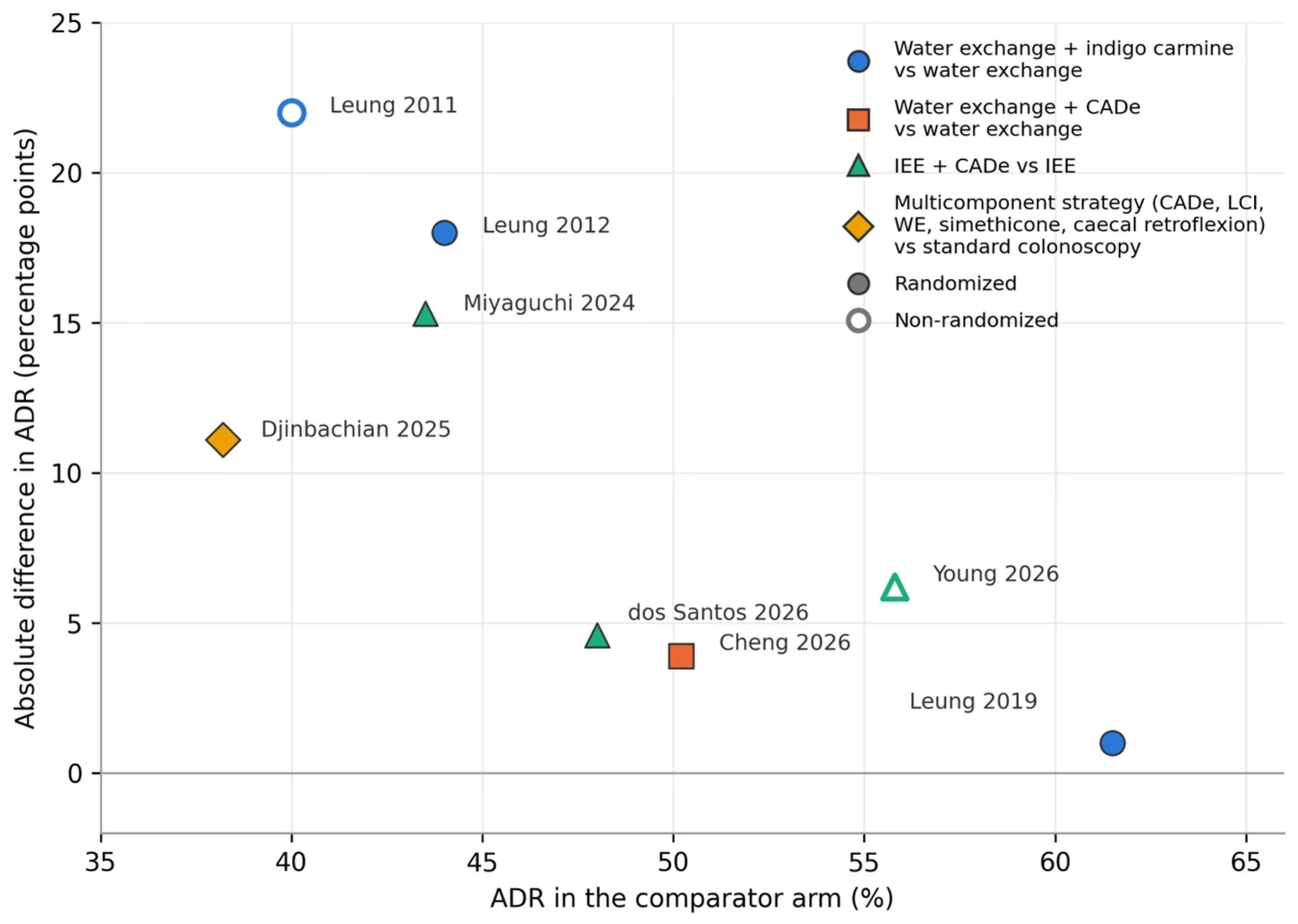
Comparator-arm adenoma detection rate (ADR) versus the absolute between-arm ADR difference in eight incremental or multicomponent comparisons. Filled markers indicate randomized studies; open markers indicate non-randomized studies. The axes are mathematically coupled because the vertical-axis difference subtracts the comparator ADR. This exploratory cross-study display does not establish a ceiling effect or identify which endoscopists benefit more. CADe, computer-aided detection; IEE, image-enhanced endoscopy; LCI, linked color imaging; WE, water exchange [14,16,17,18,19,20,21,22].

For eight studies reporting ADR in both arms, we plotted comparator-arm ADR against the absolute between-arm ADR difference (Figure 2). This was an exploratory description across studies, not a test of effect modification or an estimate of an endoscopist’s pre-study performance. Because the difference on the vertical axis subtracts the comparator ADR shown on the horizontal axis, the axes are mathematically coupled. This can contribute to an inverse pattern even without a true interaction. We therefore fitted no trend line and drew no conclusion about which endoscopists benefit more.

### 2.7. Deviations from the Protocol

We made six modifications to the registered protocol:

1. We did not conduct a search in Embase, as the database was not available through our institution. Searches in Scopus and Web of Science and the review of reference lists cannot fully compensate for this limitation.
2. The review of reference lists identified a study on water exchange combined with indigo carmine that had not been identified using the initial search terms. We therefore added terms for dye-based chromoendoscopy to the searches in Web of Science and Scopus and conducted an additional search in PubMed.
3. After identifying a study that mentioned the CADe system only by its trade name, we conducted a second additional search in PubMed, using trade names of CADe systems.
4. On 26 September 2026, after evaluating the PubMed records and before data extraction, we expanded the criterion regarding video materials: from fully recorded colonoscopies to recorded segments of the colon and video recordings of lesions analyzed with and without CADe. We report these results separately from the clinical results, which were measured at the patient level.
5. Records from Web of Science, Scopus, and supplementary searches were initially evaluated using a single tool based on a large language model, rather than two.
6. During revision, we added a structured, domain-based critical appraisal, which had not been planned in the protocol. It informed interpretation without changing the eligibility criteria or the included study set.

### 2.8. Use of Artificial Intelligence Tools

We used Claude (Anthropic) and ChatGPT (OpenAI) to assist study selection, data extraction, and language review. A large language model tool also assisted preparation of the source-linked domain-based critical appraisal during revision. We made the final eligibility decisions, and both reviewers independently verified the data extracted for the initial review against the available source reports. We take responsibility for the content of this review.

## 3. Results

### 3.1. Study Selection

Database searches identified 644 records: 164 in PubMed, 161 in Web of Science, and 319 in Scopus. After removing 270 duplicates, we evaluated 374 records based on their titles and abstracts and excluded 351. We attempted to obtain 23 publications for further evaluation. Three conference abstracts were indexed by title only, without the abstract text, and could not be evaluated. Of the 20 remaining publications, we excluded two: one compared the techniques directly, without combining them, and the other used CADe in white light and applied IEE only for lesion characterization.

A search of ClinicalTrials.gov identified four unique records. One was the registration of an included study, one concerned the monitoring of inflammatory bowel diseases, and two described studies without published results (NCT03781648 and NCT07127471). A review of the reference lists identified one additional randomized study, but neither the full text nor its abstract was available for evaluation.

We included 18 studies, reported in 19 publications (Figure 1). Thirteen studies reported clinical outcomes at the patient level, and five assessed performance using lesions or video recordings.

### 3.2. Characteristics of the Included Studies

The 18 studies were published between 2011 and 2026; 12 of them were published in 2024 or later. Seven were conducted in Japan, two in Taiwan (one of which was in collaboration with a center in Italy), one in Vietnam, and three in the United States. The remaining studies came from Canada, Brazil, Germany, Australia, and New Zealand—one from each country.

The 13 clinical studies that reported patient-level outcomes included 5411 patients, in addition to 28,459 colonoscopies from a large retrospective cohort [23] (Table 1). Nine studies were randomized, including one involving tandem examinations [24]. The other four consisted of a retrospective review of prospectively collected data using historical controls [14], a prospective comparison with historical controls [21], a prospective non-randomized tandem study [25], and a retrospective cohort [23]. Most studies were conducted at a single centre. In two trials, all procedures were performed by a single endoscopist [17,20].

IEE and CADe were used together in eight studies, excluding the one that combined all three groups of techniques. Of these eight studies, five used linked color imaging, two used texture and color enhancement imaging, and one used narrow-band imaging [19,20,21,23,24,25,26,27]. In the retrospective cohort, texture and color enhancement imaging was used in a setting where indigo carmine was also routinely applied [23]. Three studies, all from the same research group, evaluated water exchange in conjunction with dye-based chromoendoscopy [14,16,17]. One randomized study directly tested the addition of CADe to water exchange [18], and another study evaluated a strategy that included water exchange, IEE, and CADe [22]. None of the included clinical studies used water immersion.

Seven studies compared adding a technique to one already in use with using the latter alone. Another study compared two devices for mucosal exposure, while IEE and CADe were used in both arms [26]. Three studies evaluated multi-component strategies compared with white-light examination, although their designs differed [22,24,25]. In two studies, the use of the two techniques was evaluated observationally [23,27].

**Table 1:** Characteristics of the 13 patient-level clinical studies.

Study [Ref]	Country; Centres	Design	Patients	Intervention and Comparator	Type of Comparison	Main Detection Outcome	Source Charted
Leung 2011 [14]	USA; 1	Non-randomized; retrospective review; historical controls	150	WE + indigo carmine vs. WE vs. air	Incremental	ADR 62% vs. 40% vs. 36%	Full text
Leung 2012 [16]	USA; 1	RCT	168	WE + indigo carmine vs. WE	Incremental	ADR 62% vs. 44% (*p* = 0.03)	Full text
Leung 2019 [17]	USA; 1 (single endoscopist)	RCT, three arms	480	WE + indigo carmine vs. WE vs. air	Incremental	Right-colon ADR 55.6% vs. 53.4% vs. 52.2% (NS); overall ADR 62.5% vs. 61.5% vs. 61.6% (NS); right-colon serrated lesions 26.3% vs. 23.6% vs. 11.3% (*p* = 0.002, overall comparison)	Full text
Cheng 2026 [18]	Italy, Taiwan; 2	RCT, stopped early for efficacy at interim analysis	560	WE + CADe vs. WE	Incremental	APC 1.39 vs. 1.05 (RR 1.32; 95% CI 1.14–1.54); ADR 54.1% vs. 50.2% (NS)	Full text
Miyaguchi 2024 [19]	Japan; 1	RCT	800	LCI + CADe vs. LCI	Incremental	ADR 58.8% vs. 43.5% (*p* < 0.001); APC 1.31 vs. 0.94	Full text
dos Santos 2026 [20]	Brazil; 1 (single endoscopist)	RCT	622 analysed (731 randomized)	LCI + CADe vs. LCI	Incremental	ADR 52.6% vs. 48.0% (NS)	Full text
Young 2026 [21]	Australia	Prospective, historical control	263	TXI + CADe vs. TXI	Incremental	ADR 62.0% vs. 55.8% (NS); APC 1.71 vs. 1.71	Full text
Miyaguchi 2026 [26]	Japan; 1	RCT	800	Wing-hood + LCI + CADe vs. hood + LCI + CADe	Device added to IEE + CADe	ADR 58.0% vs. 39.8% (*p* < 0.001)	Full text
Akimoto 2026 [24]	Japan; 1	Randomized tandem, proximal colon	209 analysed (232 randomized)	LCI + CADe vs. WLI	Multicomponent	Proximal AMR 20% vs. 39% (*p* = 0.001)	Full text
Tran 2025 [25]	Vietnam; 1	Prospective tandem, non-randomized, right colon	116	WLI, then LCI + CADe	Multicomponent	Additional right-colon polyp detection rate: 25.9%	Full text
Djinbachian 2025 [22]	Canada; 1	Randomized trial	467	CADe + LCI + WE with simethicone + caecal retroflexion vs. WLI	Multicomponent	ADR 49.3% vs. 38.2% (*p* = 0.016)	Full text
Schauer 2026 [27]	New Zealand	RCT of CADe; NBI at endoscopist’s discretion	776	CADe vs. standard colonoscopy; NBI used in 21% of procedures	Observational evidence for CADe with NBI	ADR 63.4% vs. 57.3% (CADe vs. standard; *p* = 0.08); adjusted OR for adenoma detection: CADe 1.38, NBI 2.00	Abstract
Nishizawa 2026 [23]	Japan; 1	Retrospective	28,459 colonoscopies	TXI, CADe and newer colonoscopes; routine indigo carmine	Observational	TXI independently associated with ADR (OR 1.20); CADe not	Abstract

ADR, adenoma detection rate; AMR, adenoma miss rate; APC, adenomas per colonoscopy; CADe, computer-aided detection; CI, confidence interval; IEE, image-enhanced endoscopy; LCI, linked color imaging; NBI, narrow-band imaging; NS, not statistically significant; OR, odds ratio; RCT, randomized controlled trial; RR, rate ratio; TXI, texture and color enhancement imaging; WE, water exchange; WLI, white-light imaging. Outcomes are listed in the order of comparison groups. In Schauer 2026, CADe was randomized but NBI was used at the endoscopist’s discretion.

The other five studies analyzed recorded procedures or lesions identified during colonoscopy (Table 2). Conducted in Taiwan, Germany, and Japan, these studies reported outcomes such as the algorithm’s sensitivity, lesion recognition, or the identification of additional polyps. We present these results separately from real-time clinical detection, which was evaluated at the patient level [28,29,30,31,32].

### 3.3. Water Exchange Combined with Dye-Based Chromoendoscopy

In a non-randomized study involving 150 patients, the adenoma detection rate (ADR) was 62% for water exchange combined with indigo carmine, compared with 40% for water exchange alone and 36% for air insufflation [14]. The same group subsequently tested the addition of a low concentration of indigo carmine (0.008%) to the water used during insertion in a randomized study involving 168 patients. The ADR increased from 44% for water exchange alone to 62% for the combination of the two techniques (*p* = 0.03) [16].

A subsequent randomized, three-arm study involving 480 patients did not confirm this benefit. The ADR in the proximal colon was 55.6% for the combination, 53.4% for water exchange alone, and 52.2% for air insufflation, with no significant differences between the arms [17]. All procedures were performed by a single endoscopist, and the ADR for air insufflation was already 61.6% for the entire colon. The authors suggested that this high detection rate may have left little room for a further increase in the ADR. The detection of proximal serrated lesions differed among the three arms—26.3%, 23.6%, and 11.3%, respectively (*p* = 0.002 for the overall comparison)—but the addition of indigo carmine did not demonstrate an advantage over water exchange alone [17].

### 3.4. Water Exchange Combined with CADe

A randomized trial directly evaluated the addition of CADe to water exchange [18]. Conducted at centers in Italy and Taiwan, the study randomized 560 patients and was stopped after an interim analysis met the predefined efficacy criterion. CADe increased the number of adenomas per colonoscopy from 1.05 to 1.39 (incidence rate ratio 1.32; 95% confidence interval: 1.14–1.54). The ADR was 54.1% with CADe and 50.2% without CADe, a difference that was not statistically significant [18]. Therefore, the study demonstrated an increase in the number of adenomas identified per colonoscopy, but not in the proportion of patients in whom at least one adenoma was found.

### 3.5. IEE Combined with CADe: Incremental Comparisons

Three studies compared the use of an electronic IEE mode with and without CADe. In a single-center randomized trial involving 800 patients, the addition of CADe to linked color imaging increased the ADR from 43.5% to 58.8% (*p* < 0.001) and the number of adenomas per colonoscopy from 0.94 to 1.31 [19]. A randomized study in Brazil, with 731 patients randomized and 622 analysed after 109 post-randomization exclusions, all examined by a single experienced endoscopist, found no significant difference in the ADR between linked color imaging with and without CADe (52.6% versus 48.0%) [20]. In a prospective study in Australia, which used historical controls, the addition of CADe to texture and color enhancement imaging did not significantly increase the ADR (62.0% vs. 55.8%); the number of adenomas per colonoscopy was 1.71 in both groups [21].

A fourth randomized study addressed a related but distinct question. Both arms used linked color imaging and CADe, but the device used for mucosal exposure differed. The ADR was higher with the wing-hood device than with a standard cap (58.0% vs. 39.8%; *p* < 0.001) [26]. This comparison evaluates the device against the background of IEE and CADe use in both arms; it does not isolate the effect of adding CADe to IEE.

### 3.6. Multi-Component Strategies Compared with White-Light Examination

Three studies evaluated combinations of techniques compared with white-light examination [22,24,25]. Because several elements varied across examinations, the results cannot be attributed to a single component.

In a randomized study with tandem examinations, 209 of 232 randomized patients were analyzed following examination of the proximal colon using linked color imaging and CADe or white-light imaging alone [24]. The adenoma miss rate was 20% for the combination and 39% for white light (*p* = 0.001); the corresponding polyp miss rates were 18% and 42% (*p* < 0.001). The miss rates for sessile serrated lesions (0% vs. 46%) and advanced adenomas (0% vs. 30%) also favored the combination, but the differences were not statistically significant [24].

A non-randomized tandem-examination study conducted in Vietnam evaluated the right colon in 116 patients, first under white light and then using linked color imaging and CADe [25]. The second examination had an additional polyp detection rate of 25.9%. Because the combined techniques were always used during the second examination, the study cannot distinguish their effect from that of re-examining the same segment.

The only study that used all three groups of techniques randomized 467 patients to either an optimized strategy or standard white-light colonoscopy [22]. The optimized strategy combined CADe, linked color imaging, water exchange with simethicone, and cecal retroflexion. The ADR was 49.3% versus 38.2% (*p* = 0.016), and the polyp detection rate was 78.2% versus 59.2%. The differences in the detection of advanced adenomas (13.1% vs. 8.0%), serrated lesions (6.6% vs. 7.1%), and the number of adenomas per colonoscopy (0.86 vs. 0.75) were not statistically significant [22]. The study demonstrates the overall effect of the strategy; the authors proposed future studies to determine the contribution of each component.

### 3.7. Observational Evidence

Two studies provided information on combinations whose components were not randomly assigned together [23,27].

In a New Zealand study in which the use of CADe was randomized, endoscopists chose whether to use narrow-band imaging, a technique applied in 21% of procedures [27]. The overall ADR was 63.4% with CADe and 57.3% without CADe (*p* = 0.08). In the multivariable analysis, both CADe (odds ratio 1.38) and narrow-band imaging (odds ratio 2.00) were associated with adenoma detection. Since narrow-band imaging was used at the endoscopist’s discretion, this analysis does not establish the incremental effect of combining it with CADe.

In a retrospective cohort study in Japan, which included 28,459 colonoscopies, indigo carmine was used routinely [23]. Texture and color enhancement imaging was independently associated with a higher ADR (odds ratio 1.20), whereas CADe was not. The non-randomized choice of imaging mode and other changes in equipment limit what can be attributed to the combination.

### 3.8. Evidence at the Level of Lesions and Video Recordings

Five studies evaluated CADe using recorded data or lesions already identified during clinical colonoscopy (Table 2) [28,29,30,31,32].

Two studies explored how examination conditions influence algorithm performance. In recorded right-colon withdrawal videos, CADe identified at least one additional polyp in 30.1% of patients examined using water exchange and in 12.3% examined using air insufflation (*p* = 0.001) [28]. False-positive alerts attributed to fecal residue were also less frequent after water exchange (1.78 vs. 2.09 per colonoscopy), as were alerts attributed to bubbles (0.53 vs. 1.25). In a separate retrospective analysis of 100 polyps, CADe recognized 89% under linked color imaging and 85% under white light at normal withdrawal speed. At faster withdrawal speed, recognition fell to 75% and 67%, respectively [31].

Three other studies examined CADe with IEE more directly. A system trained with both white-light and linked color imaging images identified all 240 polyps in the full-length LCI videos used for validation; its reported false-positive frame rate was 0.001% [29]. In another study, a system trained with IEE images achieved 83% sensitivity in a selected set of 100 non-polypoid or non-reddish lesions under linked color imaging, compared with 57% for a white-light-trained system tested under white light [30]. Because training images and the testing mode both differed, that comparison cannot isolate either factor. Finally, CADe improved lesion detection in videos under both white light and linked color imaging among trainees and experts [32]. However, only lesions correctly detected by CADe during the original examination were included, and observers viewed the videos without CADe before viewing them with CADe. Selection and viewing order therefore limit interpretation.

These studies show how the systems perform under selected conditions. They do not measure how many additional lesions would be detected by using CADe during a real-time colonoscopy.

### 3.9. Procedural Burden

Several clinical studies have reported withdrawal time. This did not differ significantly between arms in five studies [18,19,20,24,26]. However, the Australian study with a historical control group found a different result: the addition of CADe to texture and color enhancement imaging prolonged the withdrawal time by nearly one minute (*p* < 0.001) and increased the proportion of resected non-neoplastic polyps from 16.6% to 27% (*p* = 0.042) [21]. In a subgroup of this study, the ADR increased with the use of CADe among endoscopists whose initial ADR was below 55% (63% vs. 44%; *p* = 0.046), although the overall comparison did not show a significant improvement [21]. In the water-exchange study, the mean number of resected non-neoplastic lesions per colonoscopy was comparable between arms [18].

A clinical study quantified the false-positive alerts generated by CADe [18]. It reported an average of 27.3 alerts per colonoscopy when CADe was used in conjunction with water exchange. Most were triggered by the intestinal wall; colonic folds accounted for 58.9% of all alerts. The two CADe systems used in the study also differed significantly in the number of false-positive alerts generated.

One study reported patient satisfaction and willingness to repeat the procedure, with no significant differences between the arms [17]. None of the included clinical studies reported financial costs or long-term outcomes, such as colorectal cancer occurring after colonoscopy.

### 3.10. Registered Studies Without Published Results

As of the search date, 26 September 2026, we identified two registered studies with no published results. NCT07127471 was listed as currently recruiting and compares water exchange combined with CADe, with and without linked color imaging; its primary outcome is clinically significant serrated lesions [33]. NCT03781648, whose recruitment status was listed as unknown, evaluates water exchange combined with narrow-band imaging for the detection of adenomas. We did not identify any publications reporting its results [34].

### 3.11. Initial Detection and Observed Gain

Eight studies reported ADR in both arms of an incremental comparison or a comparison with a multi-component strategy and were included in Figure 2. In four studies where the ADR in the comparator arm was below 45%, the absolute difference between arms ranged from 11.1 to 22.0 percentage points [14,16,19,22]. In four other studies, with an ADR of at least 48% in the comparator arm, the difference ranged from 1.0 to 6.2 percentage points [17,18,20,21].

The apparent inverse pattern is descriptive and hypothesis-generating. Comparator-arm ADR is not an independently measured baseline characteristic, and the between-arm difference subtracts that same value, creating mathematical coupling between the axes. This may contribute to the pattern. Differences in populations, interventions, and study designs further prevent interpretation as a within-study interaction or evidence of a ceiling effect.

### 3.12. Structured Critical Appraisal

Supplementary Table S2 records the supporting methods, concerns, and reporting gaps for each study. Randomization supported the direct comparisons, but operators generally knew which techniques they were using. Histological assessment and blinded pathologists, where reported, reduce some measurement concerns but do not remove the possibility that awareness of the technique affects inspection behaviour. The water exchange–CADe trial used a prespecified interim stopping rule [18]; early stopping still warrants caution about the size and precision of the observed benefit. In the Brazilian LCI–CADe trial, 109 of 731 randomized patients were excluded and 622 were analysed; post-randomization exclusions may compromise the comparability created by allocation [20]. In the randomized tandem study, 209 of 232 randomized patients were analysed, with nine exclusions occurring after the first examination; some lesions without histology were classified optically [24].

The non-randomized studies had different limitations. Historical controls leave temporal and case-mix confounding [14,21], fixed-order tandem examination cannot separate a technique effect from a second inspection [25], and discretionary NBI or equipment selection limits causal attribution [23,27]. For the three abstract-only studies, several methodological safeguards could not be assessed [23,27,31].

Lesion- and video-level studies were limited by selected material, known lesions, or comparisons that changed more than one factor. In Kobayashi 2024, selection of lesions already correctly detected by CADe and the fixed viewing order may favour assisted performance [32]. These studies therefore inform system behaviour under the tested conditions, rather than comparative effectiveness during live colonoscopy. Single-centre and single-operator designs were considered applicability concerns separately from these sources of bias.

## 4. Discussion

### 4.1. Main Results

We identified 18 studies on the combined use of water exchange, image-enhanced endoscopy (IEE), and computer-aided detection (CADe) for the identification of colorectal neoplasia. Twelve were published in 2024 or later. The evidence focuses on the combination of IEE and CADe, particularly the use of linked color imaging. Only one study directly tested the addition of CADe to water exchange [18], one evaluated a strategy that included all three techniques [22], and none of the included clinical studies used water immersion.

The interpretation depends on the comparison and its design. Randomized incremental trials found an ADR gain in some comparisons [16,19], but not a statistically significant gain in others [17,18,20]. In the water exchange trial, CADe increased adenomas per colonoscopy without establishing an increase in the proportion of patients with at least one adenoma [18]. Randomized comparisons of multicomponent strategies found better detection or lower miss rates [22,24], but could not determine which component provided the gain. Historical-control comparisons and analyses of discretionary imaging use offer weaker grounds for causal inference [14,21,23,27]. Lesion- and video-level findings describe system performance under selected conditions and do not establish additional patient-level detection during live colonoscopy.

### 4.2. Interpretation

There is a plausible clinical rationale for combining these techniques. Water exchange cleans the mucosa during insertion, IEE enhances the contrast of lesions, and CADe draws the endoscopist’s attention to suspicious lesions during withdrawal [3,8,9]. Each technique acts on a different stage of the detection process. Our review provides some support for this rationale, although much of the evidence regarding how the techniques interact comes from analyses of recorded procedures, not from real-time clinical comparisons.

In video recordings of the right colon, CADe identified at least one additional polyp in a higher proportion of patients examined using water exchange than in those examined using air insufflation. Alerts triggered by fecal matter and bubbles were also less frequent after water exchange [28]. However, in the clinical study that combined water exchange with CADe, most false-positive alerts originated from the intestinal wall, particularly from the folds [18]. A cleaner lumen could therefore reduce alerts triggered by intestinal contents without eliminating other sources of false-positive alerts. In the same study, the withdrawal time was comparable between the arms, although an average of 27.3 false-positive alerts per CADe-assisted colonoscopy was recorded [18]. The number of alerts, taken on its own, does not indicate the extent to which they interrupt the examination.

The procedural burden also needs to be assessed alongside any detection gain. The increase in non-neoplastic resections in the historical-control TXI study [21] was not reproduced in the randomized WE trial [18], and definitions and designs differed. No included clinical study measured financial costs, and patient-reported outcomes were reported in only one trial [17]. We therefore cannot establish cost-effectiveness or decide whether the additional detection justifies extra workload, surveillance, or unnecessary intervention. More lesions detected is not, by itself, a measure of net clinical benefit.

The evidence regarding the interaction between IEE and CADe is more difficult to interpret. A system trained with white-light and linked color imaging images identified all polyps in its full-length LCI validation videos, but there was no comparison across testing modes [29]. Another video study reported higher sensitivity for a system trained with IEE images and tested under linked color imaging than for a white-light-trained system tested under white light [30]. Because training images and the testing mode both changed, this comparison cannot determine which accounted for the difference. These results support further evaluation of compatibility, rather than proving that IEE-specific training provides an incremental clinical benefit.

The Australian study raises a related clinical question. The addition of CADe to texture and color enhancement imaging did not significantly improve ADR, while withdrawal time and the resection of non-neoplastic polyps increased [21]. The authors noted that the CADe system had been trained on white-light images and proposed that its use in conjunction with texture and color enhancement imaging might have contributed to the occurrence of false-positive alerts. This explanation was not directly tested, and the use of a historical control group further limits causal interpretation. Taken together, these observations support reporting and validating CADe performance for the specific imaging modality used in the study, without assuming that a mismatch between training and deployment explains the clinical outcome.

Baseline detection performance may matter, but the present evidence cannot establish a ceiling effect. Two randomized trials conducted by single endoscopists with high ADRs found no statistically significant gain from the combinations tested [17,20]. This does not demonstrate equivalence or exclude a smaller benefit. In the Australian historical-control study, a subgroup with lower prior ADR improved with CADe, although the overall comparison did not [21]; a significant subgroup result alone does not establish an interaction. Figure 2 cannot test this hypothesis either: it compares heterogeneous studies and mathematically couples comparator ADR to the difference calculated from it. Testing effect modification would require endoscopist performance to be measured before randomization and a prespecified interaction analysis within adequately powered trials.

Our findings should also be read alongside reviews of the individual techniques. Network meta-analyses have found ADR improvements with several interventions compared with standard colonoscopy, while evidence for advanced adenomas and serrated lesions has been less consistent [6,10,11]. The combination studies do not resolve that uncertainty. A three-arm water exchange trial found a difference in proximal serrated lesion detection across arms but did not show an incremental benefit from adding indigo carmine to water exchange [17]. Other studies reported no statistically significant differences for advanced or serrated outcomes [18,22,24]. These findings may be imprecise and should not be read as proof of no effect. Although ADR is associated with cancer outcomes [1], an intervention-related gain in ADR or adenomas per colonoscopy cannot by itself establish fewer post-colonoscopy cancers or deaths. None of the included clinical studies measured those long-term benefits, an uncertainty also reflected in the CADe guideline [7].

Detection and characterization also need to be distinguished. CADe prompts possible lesions; computer-aided diagnosis (CADx) predicts their histology. Two recent meta-analyses illustrate why increased detection does not automatically simplify management. For resect-and-discard, CADx did not increase the proportion of polyps that could avoid pathological assessment, although it reduced incorrect predictions with low-certainty evidence [35]. For leave-in-situ, it did not demonstrate an incremental reduction in polypectomy or in neoplastic lesions incorrectly left in place [36]. These are CADx findings, not tests of the combinations included in our evidence map. They caution against assuming that characterization tools will reliably offset the burden of additional detection.

### 4.3. Evidence Gaps and Implications for Research

The most pressing research question is which component produces the observed benefit in multi-technique strategies. In the randomized study using tandem examinations, linked color imaging and CADe were introduced together [24]. In the Vietnamese study, the combined examination was always performed during the second inspection [25]. The study that used all three groups of techniques also included simethicone and cecal retroflexion [22]. These designs evaluate strategies that might be useful in practice but cannot isolate the contribution of each individual element. Studies that add or remove components in a planned sequence or use a factorial design could answer this question.

Several combinations remain understudied. Only one study directly evaluated the addition of CADe to water exchange [18], and none of the included clinical studies used water immersion. One registered study is evaluating water exchange in combination with CADe, with and without linked color imaging, for the detection of clinically significant serrated lesions [33]. A study combining water exchange with narrow-band imaging has also been registered, but we have not identified any published results [34].

Future trials should report outcomes beyond ADR, including adenomas per colonoscopy, advanced adenomas, serrated lesions, and miss rates, with consistent definitions. They should also report withdrawal and total procedure time, false-positive alerts, non-neoplastic resections, patient experience, and costs. When CADe is combined with IEE, the system, version, and imaging modes for which it has been validated should be specified.

**Figure 3:**
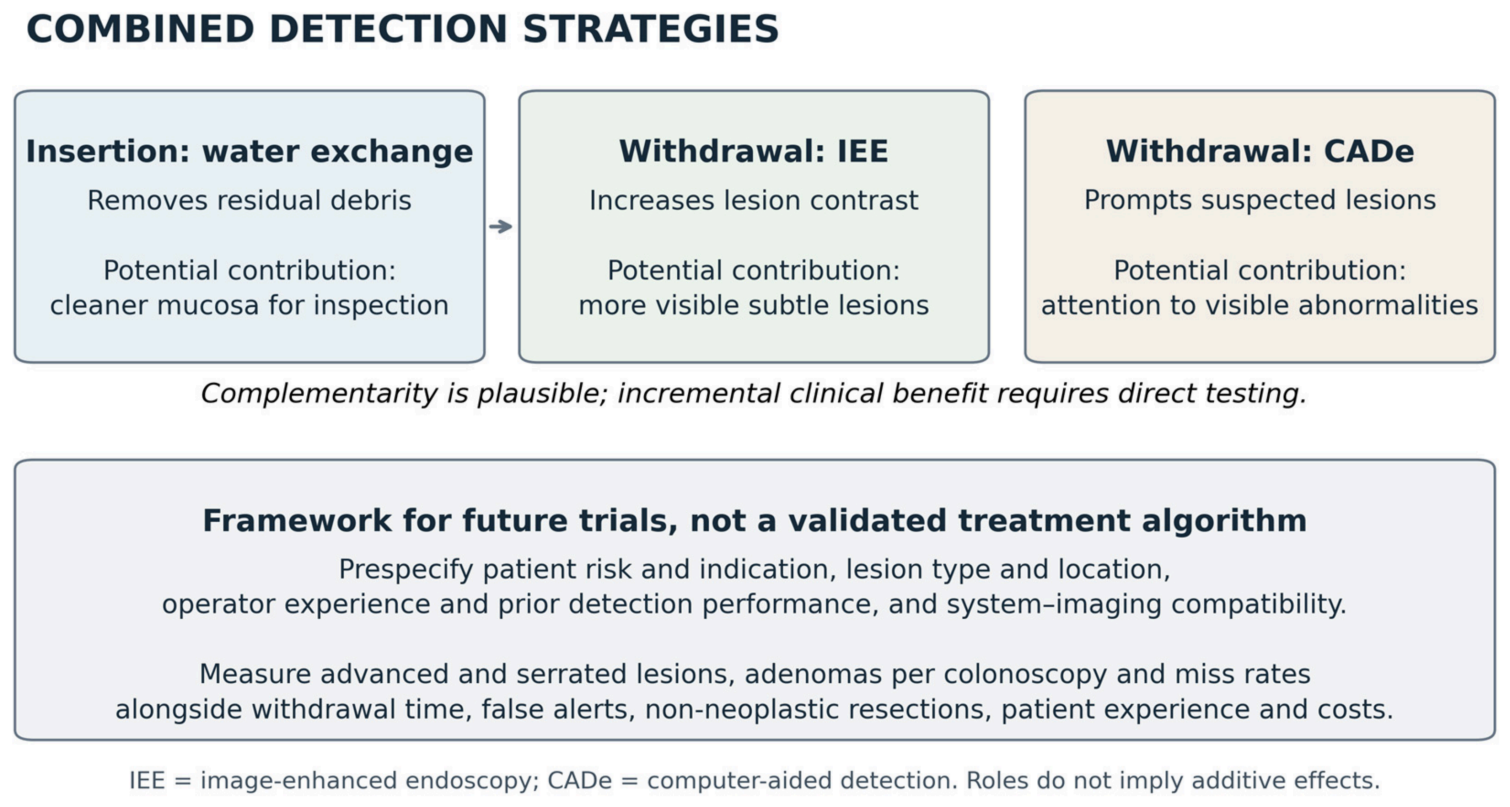
Procedural roles of water exchange, image-enhanced endoscopy (IEE), and computer-aided detection (CADe), with a proposed framework for future trials. The figure describes a clinical rationale and research priorities, not proven additive effects or a validated algorithm for selecting patients.

A framework for future subgroup analyses should consider four dimensions: patient risk and colonoscopy indication; lesion characteristics; operator performance; and technical conditions (Figure 3). Trials could prespecify analyses by screening, positive fecal testing, or surveillance, and record previous neoplasia, lesion size, morphology, histology, and proximal location. Operator experience and ADR measured before trial entry should be examined separately; expertise is not interchangeable with a contemporaneous comparator-arm ADR. Bowel preparation and the CADe–imaging configuration should also be recorded. These are proposed research dimensions, not validated criteria for choosing a combination.

Within those strata, benefit should be assessed alongside burden, using prespecified interaction tests rather than separate significant/non-significant subgroup results. Reports should give absolute detection differences with confidence intervals and relevant denominators, together with time, alert burden, non-neoplastic resections, patient experience, and costs. This would help distinguish a gain in clinically important lesions from an increase mainly in low-risk findings, and provide a basis for future individualized decisions. Current evidence does not validate a personalized treatment algorithm.

### 4.4. Strengths and Limitations

This review brings together direct clinical comparisons and studies examining algorithm performance on recorded material. We registered the protocol, disclosed that the initial MEDLINE search and screening preceded registration, and reported subsequent deviations. We searched three bibliographic databases, a trial registry, and reference lists. We distinguished incremental comparisons from packages and patient-level outcomes from lesion- and video-level performance. The structured critical appraisal added during revision makes the limits of each design explicit. We also describe the use of large language model tools and the reviewers’ checks of the original eligibility decisions and extracted data.

Several limitations affect how comprehensively we could map the evidence. Embase was not accessible, and searching other databases cannot fully compensate for potentially missed studies, particularly conference abstracts. During revision, we obtained the full text of Leung 2011 [14]; three included studies remained available only as abstracts. Three potentially eligible title-only conference records and one trial identified through reference lists could not be assessed. Requiring an English full text or abstract may also have excluded relevant evidence. These access and language limitations constrain the completeness of the map.

Interpretation is also constrained by heterogeneity in populations, techniques, comparators, and outcome definitions. Many studies were single-centre, and two trials relied on one endoscopist, which limits applicability without necessarily implying bias. Historical controls, discretionary technique use, and selected known-lesion videos create more specific concerns about confounding or selection. Our critical appraisal uses a review-specific domain framework rather than a validated risk-of-bias instrument and does not provide a formal certainty rating. Some domains could not be assessed from abstracts or other incompletely accessible reports; these gaps are identified in Supplementary Table S2. Finally, Figure 2 is an exploratory display with mathematically coupled axes and cannot establish an interaction between baseline performance and treatment effect.

## 5. Conclusions

Evidence on the combined use of water exchange, image-enhanced endoscopy, and computer-aided detection is recent and heterogeneous. Randomized incremental comparisons showed inconsistent gains in adenoma detection, while trials of multicomponent strategies could not isolate the contribution of each technique. Non-randomized and lesion- or video-level studies provide complementary evidence, with additional limitations from confounding, selection, and indirectness. The available data do not establish which patients or endoscopists benefit most, whether gains extend to clinically important lesions, or whether they outweigh procedural burden and costs. As detection systems and endoscopic imaging evolve, updated evidence on their combined use remains necessary. Future trials should isolate component effects, measure advanced and serrated lesion detection alongside burden and long-term outcomes, and prespecify analyses using independently measured endoscopist performance.

## Figures and Tables

**Table 2:** Characteristics of the five lesion- or video-level studies.

Study [Ref]	Country; Centres	Design	Material	Combination Tested	Main Outcome	Source Charted
Tang 2022 [28]	Taiwan	Video analysis of an RCT (WE vs. air)	245 videos, right colon	CADe applied to WE vs. air insufflation videos	Additional polyp detection rate: 30.1% vs. 12.3% (*p* = 0.001); false-positive alerts per colonoscopy: feces 1.78 vs. 2.09, bubbles 0.53 vs. 1.25	Full text
Neumann 2021 [29]	Germany	Validation on full-length LCI videos, no comparator	240 polyps	CADe trained with WLI and LCI; tested under LCI	Per-lesion sensitivity 100%; false-positive frame rate 0.001%	Full text
Sugino 2024 [30]	Japan	Video test set; 2 experts, 2 trainees	100 non-polypoid or non-reddish lesions; 15 full videos	CADe trained with IEE images vs. with WLI images, under WLI, BLI and LCI	Sensitivity 83% (IEE-trained CADe, LCI) vs. 57% (WLI-trained CADe, WLI) (*p* < 0.01)	Full text
Yoshida 2021 [31]	Japan; 1	Retrospective, lesions from clinical colonoscopies	100 polyps, 25 patients	CADe under WLI vs. LCI	Recognition 85% vs. 89% (normal withdrawal); 67% vs. 75% (fast withdrawal)	Abstract
Kobayashi 2024 [32]	Japan; 1	Lesion video sets with and without CADe; 3 experts, 3 trainees	100 lesions ≤ 10 mm	CADe on vs. off, under WLI and LCI	Detection without vs. with CADe, trainees: WLI 78.7% vs. 96.7%, LCI 91.3% vs. 97.3%; experts: WLI 85.3% vs. 99.0%, LCI 92.6% vs. 99.3%	Full text

BLI, blue light imaging; CADe, computer-aided detection; IEE, image-enhanced endoscopy; LCI, linked color imaging; RCT, randomized controlled trial; WE, water exchange; WLI, white-light imaging. Video- and lesion-level outcomes are not patient-level detection gains during live colonoscopy. In Kobayashi 2024, only lesions correctly detected by CADe during the live examination were included in the video sets.

## Data Availability

No new primary data were generated and that the supporting information is available in the Supplementary Materials.

## Supplementary Materials

The following supporting information can be downloaded at: https://www.mdpi.com/article/10.3390/cancers18213553/s1, Table S1: Search strategies, dates, and record counts. Table S2: Structured critical appraisal and limits of inference.
