# Supplementary material for: Beyond White-Light Colonoscopy: Water Exchange, Image-Enhanced Endoscopy, and Artificial Intelligence for Colorectal Neoplasia Detection—From Single Tools to Combined Strategies: A Scoping Review
Source: Cancers (Basel). 2026 Nov 5;18(21):3553. doi: 10.3390/cancers18213553 (PMC13761528; doi:10.3390/cancers18213553)
Supplement: Supplementary file 1 [file cancers-18-03553-s001.zip › cancers-4642692-supplementary.pdf]

Supplementary Materials

Table S1: Search strategies, dates and record counts. Table S2: Structured critical appraisal and limits of inference.

Supplementary Table S1. Search strategies, dates and number of records retrieved from each source.

Source	Search	Date	Search string	Records
MEDLINE (PubMed)	Search 1: water-aided colonoscopy + CADe	25 Sep 2026	(("water exchange" OR "water-aided" OR "water-assisted" OR "water immersion") AND ("artificial intelligence" OR "computer-aided detection" OR "computer-assisted detection" OR CADe OR "deep learning" OR "machine learning")) AND colonoscop* AND (adenoma* OR polyp* OR neoplas* OR serrated)	12
MEDLINE (PubMed)	Search 2: IEE + CADe	25 Sep 2026	(("image-enhanced endoscopy" OR "narrow band imaging" OR "narrow-band imaging" OR "linked color imaging" OR "linked colour imaging" OR "blue light imaging" OR "blue laser imaging" OR "texture and color enhancement" OR "i-scan" OR "virtual chromoendoscopy" OR chromoendoscopy) AND ("artificial intelligence" OR "computer-aided detection" OR "computer-assisted detection" OR CADe OR "deep learning" OR "machine learning")) AND colonoscop* AND (adenoma* OR polyp* OR neoplas* OR serrated)	121
MEDLINE (PubMed)	Search 3: water-aided colonoscopy + IEE	25 Sep 2026	(("water exchange" OR "water-aided" OR "water-assisted" OR "water immersion") AND ("image-enhanced endoscopy" OR "narrow band imaging" OR "narrow-band imaging" OR "linked color imaging" OR "linked colour imaging" OR "blue light imaging" OR "blue laser imaging" OR "texture and color enhancement" OR "i-scan" OR "virtual chromoendoscopy" OR chromoendoscopy)) AND colonoscop* AND (adenoma* OR polyp* OR neoplas* OR serrated)	16
MEDLINE (PubMed)	Supplementary search 1: dye-based chromoendoscopy terms	26 Sep 2026	Water-aided colonoscopy terms ("water exchange" OR "water-aided" OR "water-assisted" OR "water immersion") combined with dye-based chromoendoscopy terms ("indigo carmine" OR indigocarmine OR "dye spray") and colonoscopy terms. The exact query string was not recorded; all three retrieved records were duplicates of the main search.	3
MEDLINE (PubMed)	Supplementary search 2: CADe trade names	26 Sep 2026	("CAD EYE" OR CADEYE OR "ENDO-AID" OR "GI Genius" OR EndoBRAIN OR EndoScreener OR "WISE VISION") AND (colon* OR colorectal OR colonoscop*) AND ("linked color" OR "linked colour" OR LCI OR "blue light" OR BLI OR "narrow band" OR NBI OR "texture and color" OR TXI OR chromoendoscop* OR "indigo carmine" OR "water exchange" OR "water immersion" OR "water-aided")	12
Web of Science Core Collection	Advanced Search, topic field (TS)	26 Sep 2026	TS=(((("water exchange" OR "water-aided" OR "water-assisted" OR "water immersion") AND (("image-enhanced endoscopy" OR "narrow band imaging" OR "narrow-band imaging" OR "linked color imaging" OR "linked colour imaging" OR "blue light imaging" OR "blue laser imaging" OR "texture and color enhancement" OR "i-scan" OR "virtual chromoendoscopy" OR chromoendoscopy OR "indigo carmine" OR indigocarmine OR "dye spray") OR ("artificial intelligence" OR "computer-aided detection" OR "computer-assisted detection" OR CADe OR "deep learning" OR "machine learning"))) OR (("image-enhanced endoscopy" OR "narrow band imaging" OR "narrow-band imaging" OR "linked color imaging" OR "linked colour imaging" OR "blue light imaging" OR "blue laser imaging" OR "texture and color enhancement" OR "i-scan" OR "virtual chromoendoscopy" OR chromoendoscopy OR "indigo carmine" OR indigocarmine OR "dye spray") AND ("artificial intelligence" OR "computer-aided detection" OR "computer-assisted detection" OR CADe OR "deep learning" OR "machine learning"))) AND colonoscop* AND (adenoma* OR polyp* OR	161

Source	Search	Date	Search string	Records
			neoplas* OR serrated))	
Scopus	Title, abstract and keywords (TITLE-ABS-KEY)	26 Sep 2026	TITLE-ABS-KEY(((("water exchange" OR "water-aided" OR "water-assisted" OR "water immersion") AND (("image-enhanced endoscopy" OR "narrow band imaging" OR "narrow-band imaging" OR "linked color imaging" OR "linked colour imaging" OR "blue light imaging" OR "blue laser imaging" OR "texture and color enhancement" OR "i-scan" OR "virtual chromoendoscopy" OR chromoendoscopy OR "indigo carmine" OR indigocarmine OR "dye spray") OR ("artificial intelligence" OR "computer-aided detection" OR "computer-assisted detection" OR CADe OR "deep learning" OR "machine learning"))) OR (("image-enhanced endoscopy" OR "narrow band imaging" OR "narrow-band imaging" OR "linked color imaging" OR "linked colour imaging" OR "blue light imaging" OR "blue laser imaging" OR "texture and color enhancement" OR "i-scan" OR "virtual chromoendoscopy" OR chromoendoscopy OR "indigo carmine" OR indigocarmine OR "dye spray") AND ("artificial intelligence" OR "computer-aided detection" OR "computer-assisted detection" OR CADe OR "deep learning" OR "machine learning"))) AND colonoscop* AND (adenoma* OR polyp* OR neoplas* OR serrated))	319
ClinicalTrials.gov	Three searches	26 Sep 2026	Searches combining colonoscopy with water exchange, image-enhanced endoscopy and computer-aided detection terms. Exact query terms were not recorded. Records identified: NCT06281392, NCT01607255, NCT03781648, NCT07127471.	5 (4 unique)

Embase could not be searched because it was not available through our institution. No date or language limits were applied. After removal of duplicates across databases, 374 unique records were screened (see Figure 1). CADe, computer-aided detection; IEE, image-enhanced endoscopy.

Supplementary Table S2

Structured critical appraisal and limits of inference

We added this appraisal during peer-review revision. The review-specific framework records reported safeguards, design-specific concerns, and information that could not be assessed. It does not apply a validated risk-of-bias checklist, assign an overall risk category, or provide a numerical quality score. Absence of reporting is not treated as proof that a safeguard was absent. Single-centre and single-operator design are considered separately as applicability limitations.

Domains: selection and allocation; comparability and confounding (including co-interventions and order); outcome assessment and completeness/reporting; and the limits of the inference supported by the study. For lesion/video studies, training/testing conditions, selected known lesions, and the distinction from live clinical detection are also considered. Source locators identify the report used for each observation. Original reference numbers are retained.

Source access: Leung 2011 was retrieved in full during revision. Full texts were available for Tang 2022 and Tran 2025. Schauer 2026, Nishizawa 2026 and Yoshida 2021 remained abstract-only.

S2A Patient-level clinical studies

Study and source	Selection and allocation	Comparability and confounding	Assessment and reporting	Limits of inference and applicability
Leung 2011 [14]<br>Full text: Methods, Results and Tables 1–3; doi:10.4161/jig.1.2.16827.	Retrospective review of prospectively collected data; the combination was compared with historical WE and air groups. No random allocation.	Historical controls leave differences in time, practice and case mix as potential confounders. Reported baseline comparisons do not establish exchangeability.	Detection outcomes were reported for the three groups. Outcome-assessor masking and a prespecified analysis plan were not established from the report.	An association with detection, not a randomized incremental effect. Single-centre Veterans Affairs population limits applicability.
Leung 2012 [16]<br>Full text: Methods (randomization and sample size), Results; doi:10.4161/jig.23728.	Computer-generated allocation and sealed opaque envelopes were reported; 168 patients were assigned to WE with or without indigo carmine.	The randomized comparison changes the dye component while retaining WE. Operators could see the intervention; this may affect inspection behaviour.	ADR and the analysed group sizes were reported. Independent masking of outcome assessment was not clearly established from the accessible report.	Direct randomized evidence for the added dye under the study conditions. The small single-centre sample limits precision and generalizability.

Study and source	Selection and allocation	Comparability and confounding	Assessment and reporting	Limits of inference and applicability
Leung 2019 [17]<br>Full text: Methods (randomization, masking and outcomes), Results; doi:10.1177/2050640619832196.	Computer-generated allocation and sealed opaque envelopes were reported for the three groups.	Randomization supports the between-arm comparisons, but the endoscopist necessarily knew the insertion technique. A single operator performed all procedures.	Primary proximal ADR and serrated outcomes were reported. Patient masking and blinded collection of patient-reported outcomes do not blind the endoscopist to lesion detection.	Randomized evidence in an expert, predominantly male Veterans Affairs population. No significant ADR difference is not proof of equivalence; the overall serrated comparison does not establish an added dye effect.
Cheng 2026 [18]<br>Full text: Methods (randomization, outcomes, statistical analysis), interim analysis and supplementary material; doi:10.1055/a-2681-5544.	Computer-generated, stratified block randomization and sealed opaque envelopes were reported. The interim stopping criterion was prespecified.	WE was used in both arms; CADe was the added component. Operators were unblinded and experienced in WE. Two systems were used across two centres.	Pathologists were blinded and intention-to-treat analysis was reported. The trial stopped at the interim analysis with 560 participants; early stopping requires caution about effect magnitude and precision.	Direct randomized evidence for APC, the primary outcome. The non-significant ADR comparison does not establish equal ADR or demonstrate a cancer-prevention benefit.
Miyaguchi 2024 [19]<br>Full text: Patients and methods (randomization and analysis), Results; doi:10.1055/a-2239-8145.	Computer-generated block allocation and sealed envelopes were reported. Allocation was revealed before withdrawal.	LCI was used in both arms, isolating the added CADe comparison. Operators were aware of CADe during inspection; experts and trainees participated.	Intention-to-treat analysis and group results were reported. Independent masking of histological outcome assessment was not clearly established from the accessible report.	A direct randomized ADR comparison, with the limitations of an unblinded procedure at one centre. Operator mix and local baseline performance matter for applicability.
dos Santos 2026 [20]<br>Full text: Methods (randomization and masking), Results; doi:10.5946/ce.2025.276.	Computer-generated randomization and sealed envelopes were reported; allocation was revealed before withdrawal.	LCI was retained in both arms. Patients and pathologists were blinded, while the single expert endoscopist was not.	ADR was histologically based. Of 731 randomized patients, 109 were excluded after allocation (62 LCI; 47 LCI–CADe), leaving 622 analysed (304 and 318). Source: Results and Figure 2 (pp. 266–268).	Direct randomized comparison under one expert operator, with post-randomization exclusions that may compromise comparability; the analysed sample was not the full randomized population. A non-significant ADR difference does not establish equivalence or performance in less experienced operators.

Study and source	Selection and allocation	Comparability and confounding	Assessment and reporting	Limits of inference and applicability
Young 2026 [21] Full text: Methods (study design and control cohort), Results and Discussion; doi:10.1111/jgh.70522.	Prospective CADe cohort compared with historical TXI controls; no random assignment to the combination.	Use of the same endoscopists does not eliminate calendar-time, case-mix or practice differences. Historical control selection limits causal interpretation.	APC, ADR, withdrawal time and non-neoplastic resections were reported. The baseline-ADR subgroup finding does not itself establish an interaction.	An incremental comparison with residual confounding. The proposed imaging/training mismatch was an explanation, not an experimentally isolated mechanism.
Miyaguchi 2026 [26] Full text: Methods (allocation, masking and analysis), Results; doi:10.1055/a-2905-8041.	Random assignment using sealed opaque envelopes was reported. The sequence-generation procedure was not clearly specified in the accessible methods.	LCI and CADe were shared by both arms; only the exposure device differed. Patients and pathologists were blinded; operators were not.	All randomized participants were included in the reported analysis. Baseline imbalances were present; adjusted analyses were also reported.	Randomized evidence for the device on a shared LCI–CADe background. It cannot quantify the incremental effect of CADe or establish synergy between LCI and CADe.
Akimoto 2026 [24] Full text: Methods (randomization, tandem procedures and lesion classification), flow diagram and sensitivity analyses; doi:10.1016/j.gie.2026.04.014.	Randomized examination order was reported. Allocation concealment was not clearly established from the accessible description.	The same endoscopist performed both passes and knew the technique. Randomized order reduces, but does not remove, concerns about tandem inspection and recall.	Of 232 randomized participants, 209 were analysed after 23 exclusions; nine were excluded after the first examination. Some lesions lacked histology and were classified optically; sensitivity analyses were reported. Source: Results, participant flow and lesion-classification methods.	Randomized proximal miss-rate evidence for an LCI–CADe package. Exclusions after allocation, including after the first pass, and lesion classification warrant caution; individual component effects are not isolated.
Tran 2025 [25] Full text: Patients and methods, Results; doi:10.1016/j.ajg.2025.09.016.	Prospective, non-randomized tandem study of 116 patients with colon polyps; WLI was always followed by LCI with CADe in the right colon.	Technique and examination order are inseparable. Within-patient comparison does not control for the effect of a second inspection.	Polyps detected on the first pass were left in place and removed during the second pass for histological examination. Blinding of the endoscopist was not possible, and a prespecified analysis plan was not reported.	Additional detection on a second pass in a population selected for having polyps, not an isolated effect of LCI–CADe.
Djinbachian 2025 [22] Full text: Methods (allocation, masking and factorial design), Results and flow diagram; doi:10.1007/s00464-024-11466-7.	Computer-generated variable-block randomization, independent allocation and blinded patients/pathologists were reported.	The relevant comparison changes CADe, LCI, WE with simethicone and cecal retroflexion together. Operators were unblinded.	No post-randomization exclusions or losses were reported. Histological outcomes and group results were available.	Randomized evidence for the package as a whole. The broader factorial study design does not disentangle the components within this package.

Study and source	Selection and allocation	Comparability and confounding	Assessment and reporting	Limits of inference and applicability
Schauer 2026 [27]<br>Published abstract only;<br>doi:10.1111/jgh.70145.	CADe was randomized, but NBI was used at the endoscopist's discretion.	The NBI association is observational. Adjustment does not establish an incremental combination effect or remove all operator and patient confounding.	The abstract reports ADR and adjusted associations. Allocation concealment, masking, exclusions and missing-data handling could not be adequately assessed.	The randomized CADe result and the discretionary NBI association answer different questions. No causal interaction between NBI and CADe is established.
Nishizawa 2026 [23]<br>Published abstract only;<br>doi:10.1080/00365521.2026.2731958.	Retrospective cohort; imaging mode, CADe use and equipment were not randomized.	Routine indigo carmine, colonoscope changes, operator selection and case mix complicate attribution. Adjusted associations remain susceptible to residual confounding.	The abstract reports ADR and a multivariable model including demographics, indication, operator, colonoscope, TXI and CADe. Outcome masking, missing-data handling and full model specification could not be adequately assessed.	A large observational association, not proof that TXI or CADe caused the reported difference. Size does not resolve confounding or limited reporting.

S2B Lesion- and video-level studies

Study and source	Selection and allocation	Comparability and confounding	Assessment and reporting	Limits of inference and applicability
Tang 2022 [28]<br>Full text: Methods (video coding and CADe analysis), Results and participant flow;<br>doi:10.1016/j.gie.2021.12.020.	CADe was applied offline to 245 coded right-colon withdrawal videos from a randomized trial of WE versus air insufflation; videos with incomplete recordings or failed caecal intubation were excluded.	Randomization concerned the insertion method, not CADe use; CADe was not used during the live examinations. Two experienced endoscopists, blinded to the insertion method, reviewed the CADe-overlaid videos and identified additional polyps by consensus.	Additional polyp detection and false-positive alerts by cause were reported. Additional polyps were defined by reviewer consensus on recorded video.	Evidence about offline detection and alert sources. It does not establish a patient-level incremental benefit from live WE–CADe use.

Study and source	Selection and allocation	Comparability and confounding	Assessment and reporting	Limits of inference and applicability
Neumann 2021 [29]<br>Full text: Methods (training, validation and statistical analysis), Results; doi:10.1371/journal.pone.0255955.	Training included WLI and LCI images; validation used full-length LCI videos with 240 polyps. There was no comparison with another imaging mode.	Different polyps were reported for training and testing. A single expert assessed the validation videos; poor preparation and incomplete LCI recording were excluded.	Per-lesion detection and a false-positive frame rate were reported. Complete polyp detection in this set does not establish patient-level incremental efficacy.	Performance on a selected validation set. No comparative estimate isolates the effect of LCI or the training images, and no live ADR gain is measured.
Sugino 2024 [30]<br>Full text: Methods (video construction, systems and observers), Results and Limitations; doi:10.23922/jarc.2023-070.	Experts selected 100 non-polypoid or non-reddish lesions for matched short videos; 15 full videos were used for false-positive assessment.	The 83%-versus-57% comparison changes both training data and testing mode. Known-lesion selection limits the range of cases represented.	Sensitivity of 83% under LCI applied to the selected 100-lesion test set, not exclusively to non-polypoid lesions; 62% were polypoid (Results, Tables 1–2). Selected positive clips cannot establish patient-level detection or performance in an unselected screening population.	Mechanistic and performance evidence with spectrum and selection limitations; the reported cross-system comparison does not isolate a training effect.
Yoshida 2021 [31]<br>Published abstract only; doi:10.1007/s00384-021-04006-5.	Retrospective analysis of 100 small polyps in 25 patients, assessed under WLI and LCI at different withdrawal speeds.	Analysis of already identified lesions cannot measure how many additional lesions are found in a whole examination. Selection and testing order were not adequately described in the abstract.	Recognition rates were available and all polyps underwent histopathological examination. Reader masking and complete participant/lesion flow could not be adequately assessed from the abstract.	Lesion-level recognition under selected conditions; limited reporting precludes a comprehensive assessment of methodological safeguards.
Kobayashi 2024 [32]<br>Full text: Methods (patients, movie construction and evaluation), flow of excluded lesions and Limitations; doi:10.23922/jarc.2023-041.	The set retained 100 lesions after excluding, among others, lesions not correctly detected by CADe under the required conditions.	Selection favours CADe-detectable lesions. Observers viewed videos without CADe first and with CADe afterwards, allowing recall or learning to contribute.	Three experts and three trainees evaluated the material. The selected known-lesion set does not reproduce an unselected clinical examination.	Performance in an enriched video set, with selection and order concerns. It cannot establish incremental live ADR or unbiased sensitivity across all lesions.

Abbreviations: ADR, adenoma detection rate; APC, adenomas per colonoscopy; CADe, computer-aided detection; IEE, image-enhanced endoscopy; LCI, linked color imaging; NBI, narrow-band imaging; TXI, texture and color enhancement imaging; WE, water exchange; WLI, white-light imaging.
